# Randomised, double-blind, placebo-controlled crossover study of single-dose guanfacine in unilateral neglect following stroke

**DOI:** 10.1136/jnnp-2017-317338

**Published:** 2018-02-07

**Authors:** Edwin S Dalmaijer, Korina M S Li, Nikos Gorgoraptis, Alexander P Leff, David L Cohen, Andrew D Parton, Masud Husain, Paresh A Malhotra

**Affiliations:** 1 Department of Experimental Psychology, University of Oxford, Oxford, UK; 2 Centre for Restorative Neuroscience, Division of Brain Sciences, Imperial College London, London, UK; 3 Institute of Cognitive Neuroscience, University College London, London, UK; 4 Department of Brain Repair and Rehabilitation, Institute of Neurology, University College London, London, UK; 5 Hyper-acute Stroke Unit, Northwick Park Hospital, London, UK; 6 Division of Psychology, Department of Life Sciences, Brunel University, Uxbridge, UK; 7 Nuffield Department of Clinical Neurosciences, University of Oxford, Oxford, UK

## Abstract

**Objective:**

Unilateral neglect is a poststroke disorder that impacts negatively on functional outcome and lacks established, effective treatment. This multicomponent syndrome is characterised by a directional bias of attention away from contralesional space, together with impairments in several cognitive domains, including sustained attention and spatial working memory. This study aimed to test the effects of guanfacine, a noradrenergic alpha-2A agonist, on ameliorating aspects of neglect.

**Methods:**

Thirteen right hemisphere stroke patients with leftward neglect were included in a randomised, double-blind, placebo-controlled proof-of-concept crossover study that examined the effects of a single dose of guanfacine. Patients were tested on a computerised, time-limited cancellation paradigm, as well as tasks that independently assessed sustained attention and spatial working memory.

**Results:**

On guanfacine, there was a statistically significant improvement in the total number of targets found on the cancellation task when compared with placebo (mean improvement of 5, out of a possible 64). However, there was no evidence of a change in neglect patients’ directional attention bias. Furthermore, Bayesian statistical analysis revealed reliable evidence against any effects of guanfacine on search organisation and performance on our sustained attention and spatial working memory tasks.

**Conclusions:**

Guanfacine improves search in neglect by boosting the number of targets found but had no effects on directional bias or search organisation, nor did it improve sustained attention or working memory on independent tasks. Further work is necessary to determine whether longer term treatment with guanfacine may be effective for some neglect patients and whether it affects functional outcome measures.

**Trial registration number:**

NCT00955253.

## Introduction

Up to 80% of patients suffering from right hemisphere stroke exhibit features of unilateral neglect,^1^ a syndrome characterised by a directional attention bias away from contralesional space.^2 3^ Neglect leads to longer hospitalisation and poorer outcome,^4^ but there are no widely accepted therapies.^5–7^ It is not only a disorder of spatial attention but also comprises non-spatial attentional deficits.^8^ One of these is impaired vigilance, a reduction in the ability to sustain attention over time, which is associated with more severe neglect.^9^ In addition, phasic alerting can temporarily reduce patients’ spatial bias.^10 11^ Furthermore, the degree of sustained attention deficit correlates inversely with recovery in neglect patients,^9^ raising the possibility that neuropharmacological approaches to improving sustained attention could reduce the severity of neglect.

There is substantial evidence linking vigilance to noradrenergic pathways.^12 13^ Indeed, a small pilot study involving three right hemisphere patients with chronic neglect reported that a single dose of guanfacine (a noradrenergic alpha-2A agonist) improved space exploration in two cases.^14^ Guanfacine is licenced for the treatment of attention-deficit hyperactivity disorder (ADHD) and has positive effects on behaviour and cognition, including sustained attention.^15^ Remarkably, a single dose improves working memory in healthy humans^16^ and has also been shown to boost attention and working memory in non-human primates.^17–19^

The previous study of guanfacine in neglect^14^ employed both pen-and-paper and computerised cancellation tasks, including one that did not allow visible marking of cancelled targets-so-called invisible cancellation, which requires patients to retain the locations of previously found targets.^20^ On this task, two patients found more targets and explored more of the left side of space following guanfacine treatment when compared with placebo and baseline. They also spent more time-on-task, suggesting that guanfacine may have acted via an improvement in sustained attention. One patient with extensive prefrontal damage did not show any improvement with guanfacine, in keeping with the finding that guanfacine appears to exert its effects via alpha-2A receptors in frontal cortex.^21^ Further evidence for an effect of guanfacine on attention impairments in adult neurological patients comes from an individual with neuroinflammatory disease, whose attention deficits responded to regular guanfacine, and recurred when the drug was withdrawn.^22^

To further explore the possible beneficial effects of noradrenergic stimulation in neglect, we conducted a larger proof-of-concept, randomised, double-blind, placebo-controlled crossover investigation of the effects of a single dose of guanfacine. In this within-participant design, participants were assessed over five consecutive days. To investigate spatial exploration, we employed an invisible cancellation task. In contrast to the previous study of guanfacine in neglect, we set a fixed time limit to rule out the possibility that patients find more targets due to extended search durations. In addition, independent tests of sustained attention and working memory were administered. A secondary objective was to observe whether guanfacine might improve neglect only in patients without significant damage to prefrontal cortex.

## Methods

### Patients

Stroke patients with evidence of neglect on bedside testing were recruited from Imperial College Healthcare NHS Trust, the National Hospital for Neurology and Neurosurgery and Northwick Park Hospital. Patients were recruited during their inpatient rehabilitation or via the outpatient clinic. As in the study by Gorgoraptis and colleagues^23^ showing the effects of the dopamine agonist rotigotine on neglect, we included both acute and chronic patients. They were screened for neglect using the Mesulam shape^24^ and Behavioural Inattention Test (BIT) star^25^ cancellation tasks. Patients with robust visual neglect when tested twice with these cancellation tests (specifically an overall score on one or both tests <75% total and/or five or more omissions on the left than on the right) were considered for inclusion. Patients were initially screened for neglect up to 3 months before participation, and then carried out screening cancellation tests again immediately prior to participation to ensure that they still fulfilled the inclusion criteria. Further inclusion criteria were aged >18 years, stroke onset of at least 2 weeks prior to testing and ability to give informed consent. See table 1 for more details.

**Table 1:** Patient demographics

Subject	Age (years)	Time since stroke (moths)	Cancellation test scores at screening						Cancellation test scores at time of testing					
			BIT star			Mesulam shape			BIT star			Mesulam shape		
			Left	Right	Total	Left	Right	Total	Left	Right	Total	Left	Right	Total
**1001**	42	27	21	21	42	21	26	47	19	26	45	19	29	48
**1002**	66	3.25	0	12	12	0	11	11	0	17	17	0	10	10
**1003**	45	49	15	27	42	5	28	33	11	27	38	2	28	30
**1004**	58	14	0	14	14	0	13	13	0	18	18	0	14	14
**1005**	61	33.5	16	26	42	19	30	49	10	24	34	1	23	24
**1006**	63	2.75	14	27	41	16	15	31	26	27	53	2	29	31
**1007**	74	6	0	8	8	0	4	4	0	2	2	0	1	1
**1008**	64	1.25	8	21	29	1	26	27	25	26	51	11	24	35
**1009**	72	3	27	27	54	24	30	54	19	18	37	1	24	25
**1010**	74	6	27	27	54	16	28	44	27	24	51	7	28	35
**2001**	63	7	24	27	51	13	27	40	26	26	52	19	26	45
**2002**	75	6.5	20	26	46	8	18	26	21	14	35	8	12	20
**2003**	64	3.75	0	9	9	0	8	8	7	20	27	0	15	15

Exclusion criteria were concomitant illnesses that might affect interpretation of findings, labile blood pressure following stroke, systolic blood pressure <100 mm Hg or diastolic blood pressure <70 mm Hg, initiation of new antihypertensive medication within 3 weeks before testing, hepatic or renal dysfunction, treatment with neuroleptic medication, diagnosis of brain tumour, weight <55 kg, pregnancy or breast feeding, severe coronary insufficiency or myocardial infarction in the 6 months prior to testing, dysphasia, dementia or any other cognitive or physical impairment that would prevent a patient from providing consent or performing standard clinical tests for neglect.

**Figure 1:**
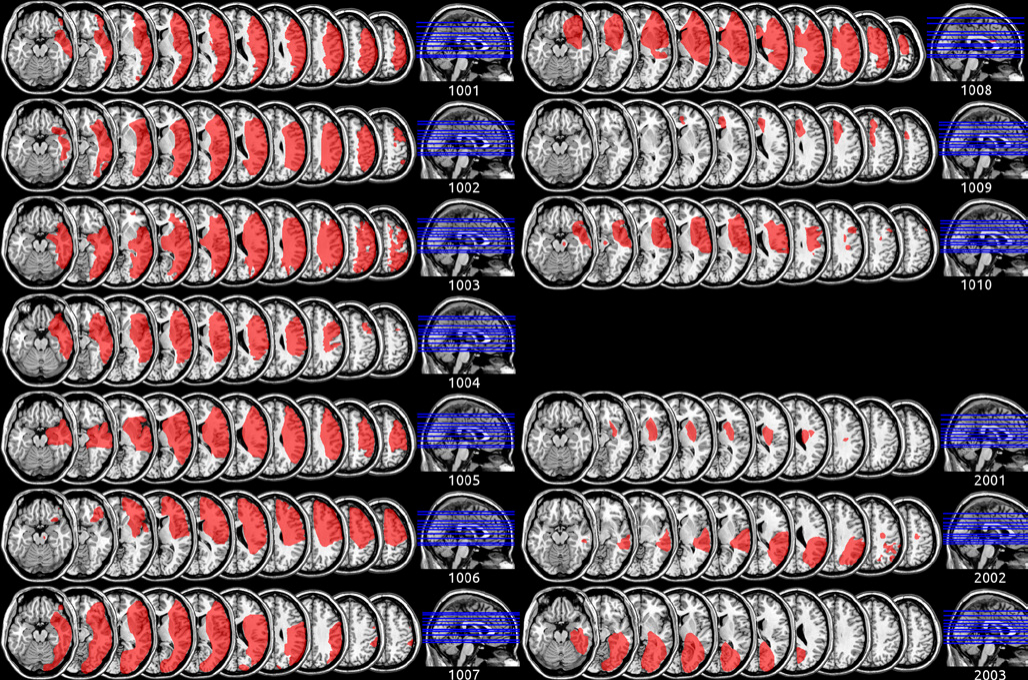
Individual lesion maps for all 13 participants. Patients 1001–1010 had some degree of cortical frontal involvement, whereas patients 2001–2003 did not.

Recruitment was terminated after expiry of the drug and placebo supply, at which point a total of 13 patients had been recruited, 10 of whom had frontal cortical damage of varying severity (figure 1A) and 3 of whom had no significant frontal cortical damage (figure 1B). Table 1 provides an overview of patient demographics and test scores at the time of inclusion.

### Procedure

A stratified crossover protocol was employed, allowing for within-patient comparison between treatment and placebo, while removing between-patient variability. Oral guanfacine (2 mg; as in refs 14 16) was encapsulated by Nova Pharmaceuticals, which also provided matching placebo.

Patients were tested on five consecutive days. On days 1, 3 and 5, they were tested on a task battery that consisted of a computerised ‘invisible’ touchscreen cancellation task,^20^ plus independent tests of sustained attention^26^ and spatial working memory (further details below).

On day 2, individuals received active drug or placebo, and on day 4 they received placebo if they had previously received active drug and vice versa. The order of administration of guanfacine and placebo was counterbalanced across patients, according to a pregenerated randomisation scheme. The clinician who administered the drugs and the tests was blind to the randomisation and the drug. The analyses presented here were performed by a different researcher, only after all data were collected. On both treatment days, patients were tested on the task battery twice: once immediately before guanfacine/placebo administration and once 2 hours after. As guanfacine is washed out within ~17 hours, residual effects were not expected to be present by day 4 if individuals had received guanfacine on day 2. Patients reported no side effects.

All patients provided informed written consent.

### Touchscreen cancellation

Standard cancellation tasks require participants to search for and mark targets, leaving a visible ‘cancellation’ of each marked target. We employed a touchscreen search task that allowed patients to touch targets *without* marking them^14 20^. Such an invisible cancellation procedure has been shown to be more sensitive to neglect than tests with visible markings.^27^ We used three different arrays that were matched in the number and distribution of targets (64) and distractors (128) to prevent learning of target locations in each array. Each patient saw each version only twice and never in direct succession. The order of arrays was randomised between patients. A strict time limit of 2 min was enforced.

As well as recording the total number of targets found, we obtained indices of patients’ directional bias and search organisation by employing CancellationTools, a freely available software package.^28^ This provides nearly all previously reported cancellation indices and has been used in contemporary neuropsychological research. As a measure of *general performance*, we computed the total number of cancelled targets. To examine *directional bias* in spatial attention, we computed the difference in the number of cancelled targets on right and left halves of the array, as well as the centre of cancellation,^29^ which is computed as the average horizontal position of all cancelled targets (scaled so that −1 corresponds with the left-most target, and 1 with the right-most).

Although the number of cancelled targets and the directional bias were the primary measures of interest, cancellation tasks also allow the computation of additional measures. We computed the number of *revisits*. These are targets that patients cancelled more than once, either immediately by cancelling the same target multiple times in a row or delayed by returning to a target after cancelling others. In addition, *search organisation* was quantified by correlating cancellation rank order and corresponding horizontal/vertical locations, the rate of search path intersections, the absolute and standardised intercancellation distance and the standardised intercancellation angle.

### Sustained attention

We employed a paradigm in which the targets were red or green triangles that pointed downwards, while non-targets were red triangles that pointed upwards. Stimuli were presented for 1000–1500 ms and were interleaved with interstimulus intervals of 1000–1500 ms^23^. Patients had to press a button when they detected a target, but withhold from pressing when non-target stimuli were presented. In total, 320 stimuli were shown, of which 40 were green targets, 40 were red targets and the remaining 240 were non-targets. The task lasted approximately 10 min.

As the main outcome measure, we computed response time variability, which is commonly used as an index of sustained attention, with higher variability indicating poorer deployment of attention on task. In order to track patients’ sustained attention over the course of the test, we binned their correct responses into five bins. Each bin contained a minimum of five trials, and reaction time variability was calculated as the SD of all response times within a bin.

In addition, the task allowed us to compute the proportion of hits, misses, false alarms and correct rejections, as well as response sensitivity (d′) and bias (criterion (c)) in terms of signal-detection theory.^30^

### Spatial working memory

A shortened version of a vertical (non-lateralised) spatial working memory task that has previously been used to assess neglect patients^31^ was employed. In each trial, a sequence of highlighted locations (circles) was displayed. Locations could be presented at any of 10 different positions along the vertical midline of the computer screen (five above and five below a central fixation cross), but the same location was never repeated within a sequence. After observing a sequence, patients were presented with a probe display that contained nine black discs and a single highlighted location. They were required to verbally indicate whether the probed (highlighted) location was part of the sequence. Location sequences varied in length from one to five stimuli and became progressively longer, with an increase of 1 every 10 trials. Thus, trials 1–10 were of sequence length 1, trials 11–20 were of length 2, and so on until trials 41–50, which consisted of sequence lengths of 5 locations. Mean accuracy (proportion of correct responses) per sequence length was used as the variable of interest.

### Data analysis

Baseline performance was determined for each patient by averaging scores on days 1, 3 and 5, as well as the preadministration sessions on days 2 and 4. Group averages and differences were computed between treatment type (baseline, guanfacine and placebo) across individuals.

To test whether there was a systematic effect of treatment type, we employed repeated-measures analyses of variance (ANOVAs). Drug was a factor in all analyses, with three levels: baseline, guanfacine and placebo. For the sustained attention task, time bins were included as an additional factor, with five levels: one for each time bin. This allowed us to assess performance over the course of the experiment. For the spatial working memory task, sequence length was included as an additional factor, with five levels: one for each sequence length.

Traditional (frequentist) repeated-measures ANOVAs produce P values, which inform us whether the null hypothesis should be rejected or not but not how well it is supported by the data.^32^ To address this, we performed Bayesian repeated-measures ANOVAs,^33^ which produce a Bayes factor (BF_10_). This is the probability of the alternative hypothesis (‘*guanfacine changes patients’ performance*’) divided by the probability of the null hypothesis (‘*guanfacine does not change patients’ performance*’). In essence, the Bayes factor is a quantification of how much confidence one can have in either hypothesis. We interpret our results following the guidelines of Jeffreys,^34^ which considers a Bayes factor over 3 as evidence in support of the alternative hypothesis. Conversely, a Bayes factor under 1/3 would support the null hypothesis.

Data were handled in custom Python^35 36^ software, using the NumPy and SciPy libraries^37^ for computations and the Matplotlib library^38^ for plotting. All statistical analyses were performed in JASP, V.0.7.1.12.^39^ For the Bayesian analyses, a Cauchy prior of 0.707 was set (JASP default setting), and it was confirmed for each test that using a wider prior did not affect the direction or exaggerate the evidence.

## Results

### Touchscreen cancellation: general performance

**Figure 2:**
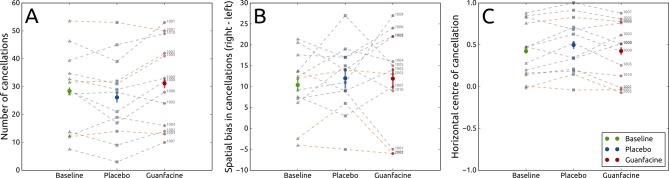
Total number of correctly marked targets in a cancellation task (A), as well as the difference between the number of correctly marked targets on the right and the left sides of the task (B) and the centre of cancellation where positive values indicate a rightward bias (C) in the baseline (green) condition and after placebo (blue) or guanfacine (red) administration. Solid horizontal lines indicate the mean, and error bars indicate within-participant 95% CIs. Each set of three connected dots represents a participant (grey for patients with cortical frontal involvement, and orange for without).

There was a significant main effect of drug on the total number of targets found, with a mean of five more targets (out of a possible 64) cancelled on guanfacine versus placebo, F(2, 24)=5.66, P=0.010, ω^2^=0.26, BF_10_=4.926. Post hoc paired-sampled t-tests revealed a significant improvement in the total number of targets found between baseline (M=28.4, SD=13.91) and guanfacine (M=31.15, SD=15.09), t(12)=−2.21, P=0.047, Cohen’s d=−0.613, BF_10_=1.687, as well as between the placebo (M=26.15, SD=14.29) and guanfacine conditions, t(12)=−2.93, P=0.013, Cohen’s d=−0.813, BF_10_=4.806. Importantly, by contrast, there was no significant difference between baseline and placebo conditions, t(12)=1.52, P=0.154, BF_10_=0.704. These results provide moderate evidence of a significant effect of drug on search performance with the total number of targets found increasing by, on average, five on guanfacine compared with placebo (figure 2A).

Qualitatively, there did not seem to be a difference between the patients with (grey lines in figures) and without (orange lines) frontal involvement on any of the metrics reported here and in the supplementary materials.

### Touchscreen cancellation: directional bias

Independent repeated-measures ANOVAs revealed no main effect of drug on the difference between the cancellations on the right and left sides of the cancellation task (figure 2B), F(2, 24)=0.39, P=0.683, BF_10_=0.231, and no main effect of drug on the centre of cancellation (figure 2C), F(2, 24)=2.45, P=0.108, BF_10_=0.848. A direct comparison of the difference in right and left cancellations in the placebo (M=12.0, SD=7.99) and guanfacine (M=11.92, SD=11.36) conditions revealed no difference between the two, t(12) = 0.03, P=0.976, BF_10_=0.278.

In sum, these results provide inconclusive evidence for an effect of drug on the centre of cancellation, but they do provide moderate evidence against an effect of guanfacine on the right minus left directional bias.

### Touchscreen cancellation: revisits and search organisation

We found no conclusive evidence for an effect of drug on revisits (online supplementary figure 1) and moderate evidence against there being a main effect of drug in all indices of search organisation (online supplementary figure 2). Exact test results and direct comparisons between guanfacine and placebo can be found in the supplementary materials.

10.1136/jnnp-2017-317338.supp1 Supplementary file 1

### Sustained attention

A repeated-measures ANOVA revealed no main effect of drug on reaction time variability (online supplementary figure 3E). F_Greenhouse-Geisser_(1.38, 16.61)=0.96, P=0.371, BF_10_=0.100, nor a main effect of time, F(4, 48)=0.20, P=0.939, BF_10_=0.022. There was also no interaction between drug and time, F(8, 96)=0.72, P=0.673. These results provide moderate to strong evidence that there was no effect of drug or time on reaction time variability in the sustained attention task.

Signal-detection variables were also computed and analysed and are reported in the supplementary materials.

### Spatial working memory

A repeated-measures ANOVA revealed no main effect of drug on response accuracy, F(2, 24)=0.84, P=0.446, BF_10_=0.082, a main effect of sequence length, F_Greenhouse-Geisser_(2.54, 30.42)=6.39, P=0.003, ω^2^=0.30, BF_10_=28 919.401 and no interaction effect, F(8, 96)=1.42, P=0.200. These results provide strong evidence that there was no effect of drug on response accuracy and decisive evidence that there was an effect of sequence length on response accuracy (with worse accuracy for longer sequences, as one might anticipate).

Post hoc paired-sampled t-tests indicated that there was no difference between baseline and guanfacine for each sequence length (all P>0.05; BF_10_ for sequence lengths 1, 3, 4 and 5 ranged between 0.358 and 0.554, and BF_10_ for sequence length 2 was 1.533) nor any difference between placebo and guanfacine for each sequence length (all P>0.05; BF_10_ ranged between 0.279 and 0.328). For sequence lengths 1–4, there was no difference between baseline and placebo (all P>0.05, BF_10_ were 1.183, 1.116, 0.464 and 0.278 for sequence lengths 1–4, respectively). In sum, there was a reliable absence of an effect of treatment type on accuracy in the spatial working memory task.

## Discussion

Here we investigated whether a single 2 mg dose of guanfacine, an alpha-2A adrenoreceptor agonist, has beneficial effects for patients who suffer from unilateral neglect. By supplementing frequentist with Bayesian statistics,^32 33^ we aimed to establish whether any null effects were due to guanfacine not being different from placebo, or simply due to a lack of statistical power. Our results indicate that guanfacine, when compared with baseline and placebo, led to a significant improvement in the total number of targets found on a touchscreen cancellation task (mean of 5 out of a possible 64 targets) in which patients have to keep track of previously found locations (figure 2). However, there was no conclusive evidence as to whether guanfacine improved directional bias. Specifically, there was moderate evidence that there was no effect of guanfacine on the difference between the number of cancellations on the right and left sides of the task, but there was no conclusive evidence for or against an effect of guanfacine on the centre of cancellation. There was also no conclusive evidence for guanfacine affecting revisits.

There was moderate evidence for the absence of an effect of guanfacine on search organisation, operationalised with four indices. In addition, we found moderate to strong evidence that guanfacine does not improve response time variability, but no conclusive evidence of whether it affects signal detection on a sustained attention task. Finally, there was strong evidence that guanfacine does not improve spatial working memory.

This study follows on directly from a previous investigation in which three neglect patients were tested on an identical but time-unlimited ‘invisible’ cancellation task.^14^ A beneficial effect of guanfacine was found for two patients without frontal involvement.^14^ This manifested as a larger number of targets found *plus* increased time-on-task. These findings could have been caused by improvement in either or both the directional bias and sustained attention components of neglect. In this larger sample of 13 neglect patients, we again found an increase in the number of targets found on the same computerised visual search paradigm following guanfacine. However, we could not demonstrate statistical evidence for a simultaneous reduction in directional bias on guanfacine. A larger study with more statistical power could potentially address this.

Although there is evidence for the beneficial effects of guanfacine on working memory in monkeys^17^ and healthy humans,^16^ we found no conclusive evidence that it reduced revisit rates on the ‘invisible’ cancellation task. In addition, there was strong evidence that guanfacine did not affect accuracy on a vertical spatial working memory task.

One interpretation of the findings of Malhotra and colleagues^14^ is that guanfacine boosted sustained attention, which caused patients to perform the task for longer, increasing the chance that they found more targets, without any direct effect on directional bias. This is partly supported by the observation that one patient whose search improved with guanfacine also showed improvement on a separate non-lateralised sustained attention test.^14^ However, we found moderate statistical evidence for the absence of an effect of treatment type on sustained attention. Given that we employed a strict time limit for the touchscreen search task, guanfacine cannot have exerted its effects by modulating time-on-task in the current study. However, it is still possible that guanfacine improved alertness and generalised arousal leading to more targets found within the time available. This might also account for the lack of any effect on lateralised bias.

Stroke patients with neglect are more likely to also suffer from disorganised search,^40 41^ and some have argued that this is a consequence of disturbed spatial attention.^42^ Across four indices of search organisation, we found moderate statistical evidence that guanfacine did not result in any improvements.

The previous study of guanfacine in neglect tentatively suggested that guanfacine’s effects might have longer lasting effects than its 17 hours wash-out period would suggest, on the basis of one patient doing relatively well on tests 1 week after guanfacine administration.^14^ No such effects appear in the current data.

The current study provides further evidence that guanfacine modulates search in neglect. However, the benefit is relatively small and appears in a highly sensitive test, leaving the exact clinical value unclear. To demonstrate a convincing role for noradrenergic therapies, it is essential to evaluate *longer term* treatment in this group, as at present, there is only single-case level evidence that regular guanfacine may be effective in reducing attentional impairment caused by neurological disease.^22^ Moreover, given that the effect we observed was relatively modest, any further study might also profitably explore the effects of increasing drug dose. In ADHD, the recommended maximum daily dose is 0.12 mg/kg compared with the total 2 mg dose used here.

Neglect is considered to be a heterogeneous syndrome, consisting of a core directional bias and additional cognitive deficits that increase clinical severity.^4 43^ As these deficits may not all respond to different therapies, and as neglect severity may vary on a day-to-day basis, novel trial designs might be particularly helpful in determining whether an intervention is efficacious^23^. In particular, we advocate the use of highly precise measures in addition to traditional clinical scales. For example, the invisible cancellation task is more sensitive to neglect symptoms and provides additional information on domains as working memory and executive functioning.^28^ This information could inform whether a drug works *and* what the underlying cognitive mechanism is. It should also be noted that a combination of (pharmacological and behavioural) interventions might prove more efficacious than a single therapy.

## Conclusion

We conducted a randomised, double-blind, placebo-controlled, crossover study examining the effects of a single 2 mg dose of guanfacine, a noradrenergic alpha-2A agonist. Thirteen stroke patients with unilateral neglect were tested on an ‘invisible’ cancellation paradigm, as well as sustained attention and spatial working memory tasks. A significant improvement in the total number of targets patients found on the cancellation task was observed on guanfacine versus placebo, but there was no evidence for or against beneficial effects of guanfacine on directional bias. Guanfacine did not improve search organisation and did not affect performance on sustained attention or spatial working memory tasks. Further work is now necessary to determine whether regular treatment with guanfacine has beneficial effects on neglect and activities of daily living.
