# Supplementary material for: Randomised, double-blind, placebo-controlled crossover study of single-dose guanfacine in unilateral neglect following stroke
Source: J Neurol Neurosurg Psychiatry. 2018 Feb 7;89(6):593–8. doi: 10.1136/jnnp-2017-317338 (PMC6031270; doi:10.1136/jnnp-2017-317338)
Supplement: Supplementary file 1 [file jnnp-2017-317338supp001.pdf]

Supplementary Materials

Additional statistical analyses and figures for Dalmaijer & Li et al., *A randomised double-blind, placebo-controlled crossover study of single-dose guanfacine in unilateral neglect following stroke*. These analyses concern secondary outcome measures that relate to non-spatial deficits that could be implicated in neglect, such as revisits and search organisation.

Supplementary Results

Touchscreen Cancellation – revisits

Independent repeated-measures ANOVAs revealed no main effect of drug on the total number of revisits in this ‘invisible’ cancellation task where items already cancelled are not left marked on the screen (**Supplementary Figure 1A**), $F(2, 24) = 1.43$, $p = 0.259$, $BF_{10} = 0.451$; no main effect of drug on the number of immediate revisits (perseverations; **Supplementary Figure 1B**), $F(2, 24) = 1.15$, $p = 0.334$, $BF_{10} = 0.387$; and no main effect of drug on the number of delayed revisits (**Supplementary Figure 1C**), $F(2, 24) = 2.37$, $p = 0.115$, $BF_{10} = 0.805$. These results provide no conclusive evidence on whether drug had an effect on revisits.

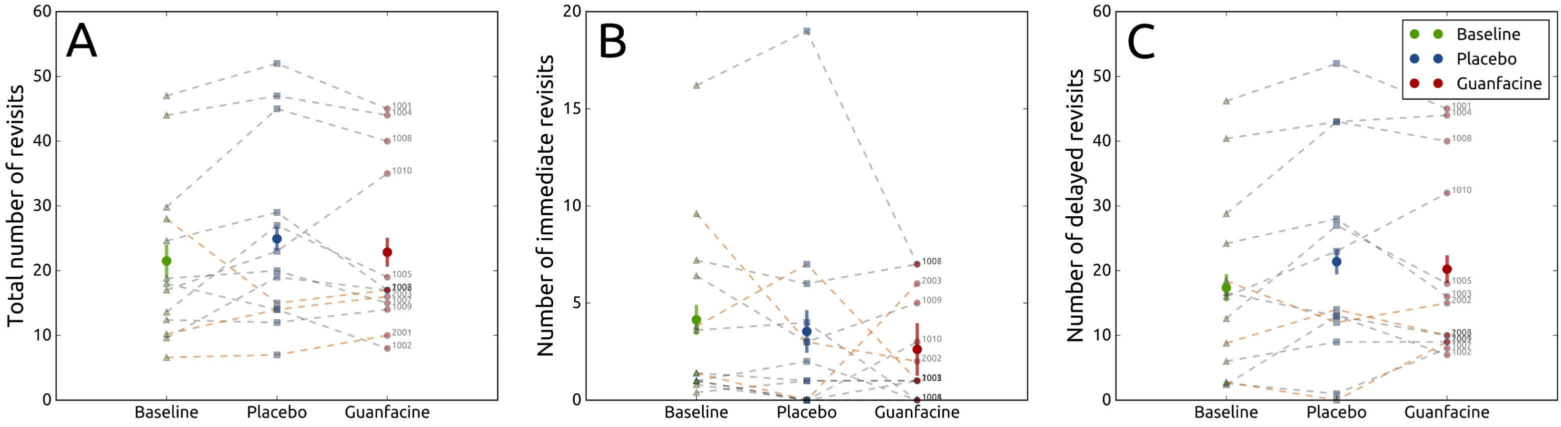

Supplementary Figure 1 – *Total number of revisits in a cancellation task (A), as well as the number of immediate revisits or perseverations (B), and the number of delayed revisits (C) in the baseline (green) condition, and after placebo (blue) or guanfacine (red) administration. Solid horizontal lines indicate the mean, and error bars indicate within-participant 95% confidence intervals. Each set of three connected dots represents a participant (grey for patients with cortical frontal involvement, and orange for without).*

Touchscreen Cancellation – search organisation

The best R was computed as the highest correlation between cancellation rank number, and either the corresponding vertical or the horizontal cancellation coordinate. Potential values range

between 0 (not organised) and 1 (organised). A repeated-measures ANOVA found no main effect of drug on the best R, $F(2, 24) = 0.37$, $p = 0.694$, $BF_{10} = 0.235$; and a paired-samples t-test found no difference in the best R between guanfacine (M = 0.51, SD = 0.29) and placebo (M = 0.49, SD = 0.26), $t(12) = 0.31$, $p = 0.760$, $BF_{10} = 0.290$. These results provide moderate evidence that drug had no effect on the best R, and that there was no difference between guanfacine and placebo (**Supplementary Figure 2A**).

The intersection rate was computed as the number of times a participant's search path intersected with itself, divided by the total number of cancellations that are not immediate revisits. Independent repeated-measures ANOVAs revealed no main effect of drug on the total number of search path intersections, $F_{Greenhouse\text{-}Geisser}(1.41, 16.86) = 0.67$, $p = 0.475$, $BF_{10} = 0.280$; and no main effect of drug on the intersection rate, $F_{Greenhouse\text{-}Geisser}(1.18, 14.10) = 0.50$, $p = 0.521$, $BF_{10} = 0.253$. Direct comparisons (paired-samples t-tests) between guanfacine and placebo revealed no difference in the total number of intersections (guanfacine: M = 18.77, SD = 24.67; placebo: M = 17.08, SD = 16.71), $t(12) = 0.35$, $p = 0.733$, $BF_{10} = 0.294$; nor in the intersections rate (guanfacine: M = 0.37, SD = 0.51; placebo: M = 0.30, SD = 0.30), $t(12) = 0.55$, $p = 0.596$, $BF_{10} = 0.317$. These results provide moderate evidence that drug had no effect on the intersections total (**Supplementary Figure 2B**) and rate (**Supplementary Figure 2C**), and that there was no difference between guanfacine and placebo.

The inter-cancellation distance was computed as the average distance (in pixels) between each cancellation and the next. The standardised inter-cancellation distance was defined as the average inter-cancellation distance in pixels, divided by the average distance between each target its nearest neighbouring target (which makes this metric comparable between different cancellation tasks). Independent repeated-measures ANOVAs revealed no main effect of drug on the average inter-cancellation distance, $F(2, 24) = 0.86$, $p = 0.435$, $BF_{10} = 0.316$; and no main effect of drug on the standardised inter-cancellation distance, $F(2, 24) = 0.48$, $p = 0.624$, $BF_{10} = 0.253$. Direct comparisons (paired-samples t-tests) between guanfacine and placebo revealed no difference in the average inter-cancellation distance (guanfacine: M = 218.4, SD = 43.58; placebo: M = 224.6, SD = 28.79), $t(12) = -0.71$, $p = 0.493$, $BF_{10} = 0.345$; nor in the standardised inter-cancellation distance (guanfacine: M = 2.45, SD = 0.47; placebo: M = 2.52, SD = 0.30), $t(12) = -0.74$, $p = 0.472$, $BF_{10} = 0.353$. These results provide moderate evidence that drug had no effect on the average (**Supplementary Figure 2D**) and standardised (**Supplementary Figure 2E**) inter-cancellation distances, and that there was no difference between guanfacine and placebo.

The standardised angle is defined as the angle between consecutive cancellations, standardised so that a value of 1 corresponds with a completely cardinal angle, and a value of 0 with a completely diagonal angle. A repeated-measures ANOVA found no main effect of drug on the

standardised angle, F(2, 24) = 0.07, p = 0.937, BF_{10} = 0.187; and a paired-samples t-test found no difference in the standardised angle between guanfacine (M = 0.66, SD = 0.075) and placebo (M = 0.65, SD = 0.11), t(12) = 0.05, p = 0.959, BF_{10} = 0.279. These result provides moderate evidence that drug had no effect of the standardised angle, and that there was no difference between guanfacine and placebo (**Supplementary Figure 2F**).

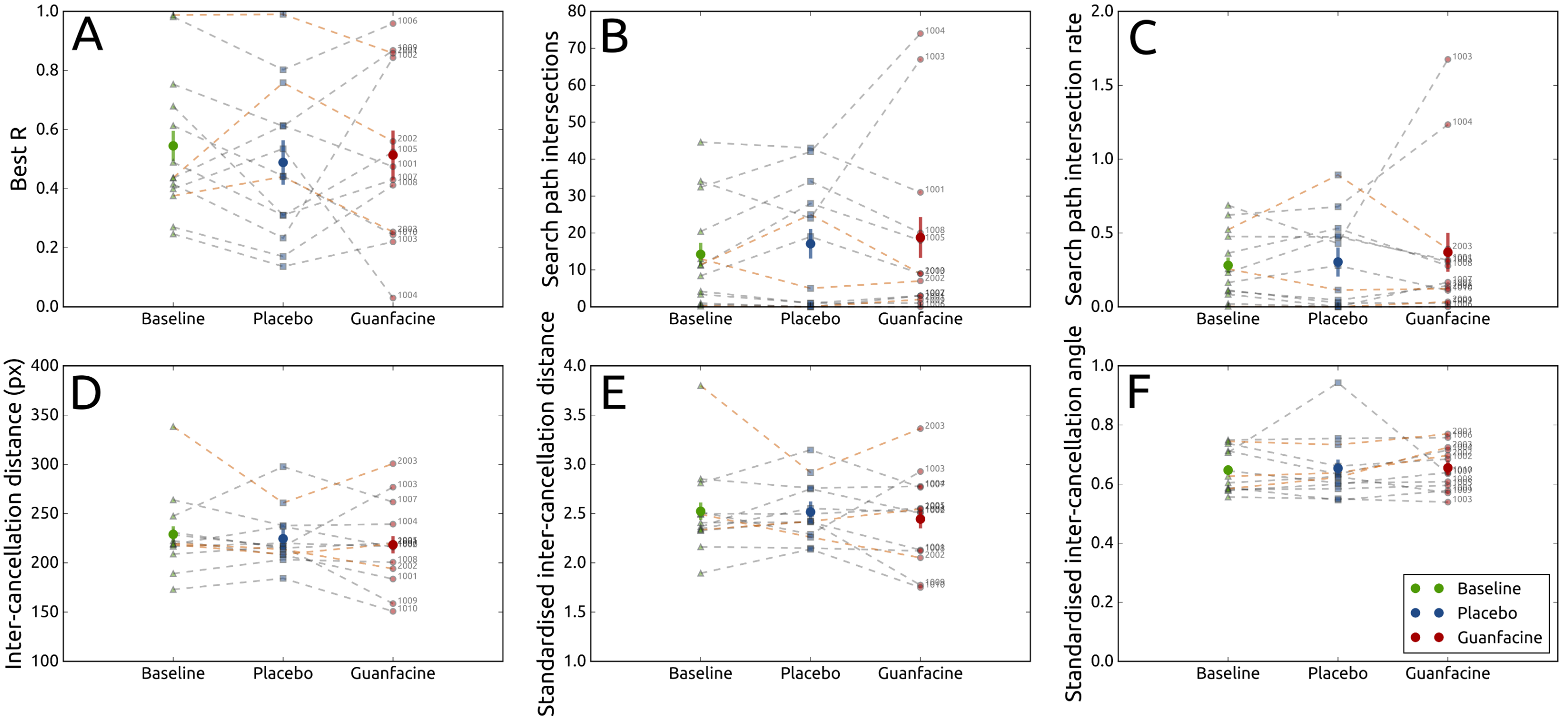

Supplementary Figure 2 – *Metrics of search organisation in a cancellation task: Best R (A), total number of search path intersections (B), search path intersection rate (C), inter-cancellation distance (D), standardised inter-cancellation distance (E), and standardised inter-cancellation angle (F) in the baseline (green) condition, and after placebo (blue) or guanfacine (red) administration. Solid horizontal lines indicate the mean, and error bars indicate within-participant 95% confidence intervals. Each set of three connected dots represents a participant (grey for patients with cortical frontal involvement, and orange for without).*

Sustained attention – signal detection

Independent repeated-measures ANOVAs revealed no main effect of drug on hit rate (**Supplementary Figure 3A**), F(2, 24) = 2.84, p = 0.078, BF_{10} = 1.061; no main effect of drug on false alarm rate (**Supplementary Figure 3B**), $F_{Greenhouse\text{-}Geisser}$(1.12, 13.39) = 1.51, p = 0.243, BF_{10} = 0.474; no main effect of drug on sensitivity (d'; **Supplementary Figure 3C**), F(2, 24) = 1.05, p = 0.367, BF_{10} = 0.359; and a main effect of drug on response bias (c; **Supplementary Figure 3D**),

F(2, 24) = 5.31, p = 0.012, ω^2 = 0.24, BF_{10} = 4.199. Post-hoc paired-samples t-tests indicated that there was no difference in response bias (c) between the baseline (M = 0.69, SD = 0.44) and guanfacine (M = 0.72, SD = 0.42) condition, t(12) = -0.47, p = 0.650, BF_{10} = 0.306; but that there was a difference in response bias (c) between the baseline and placebo (M = 0.51, SD = 0.45) conditions, t(12) = 2.73, p = 0.018, Cohen's d = 0.76, BF_{10} = 3.544; as well as between the guanfacine and placebo conditions, t(12) = 3.51, p = 0.004, Cohen's d = 0.973, BF_{10} = 11.555.

These results provide no conclusive evidence of a presence or absence of an effect of drug on hit rate, false alarm rate and sensitivity (d'). The results do provide moderate evidence that placebo reduced the response bias (c) to a less conservative value compared to baseline (values of c between 0 and 1 indicate a bias to not responding), and that guanfacine had no effect on response bias (compared to baseline).

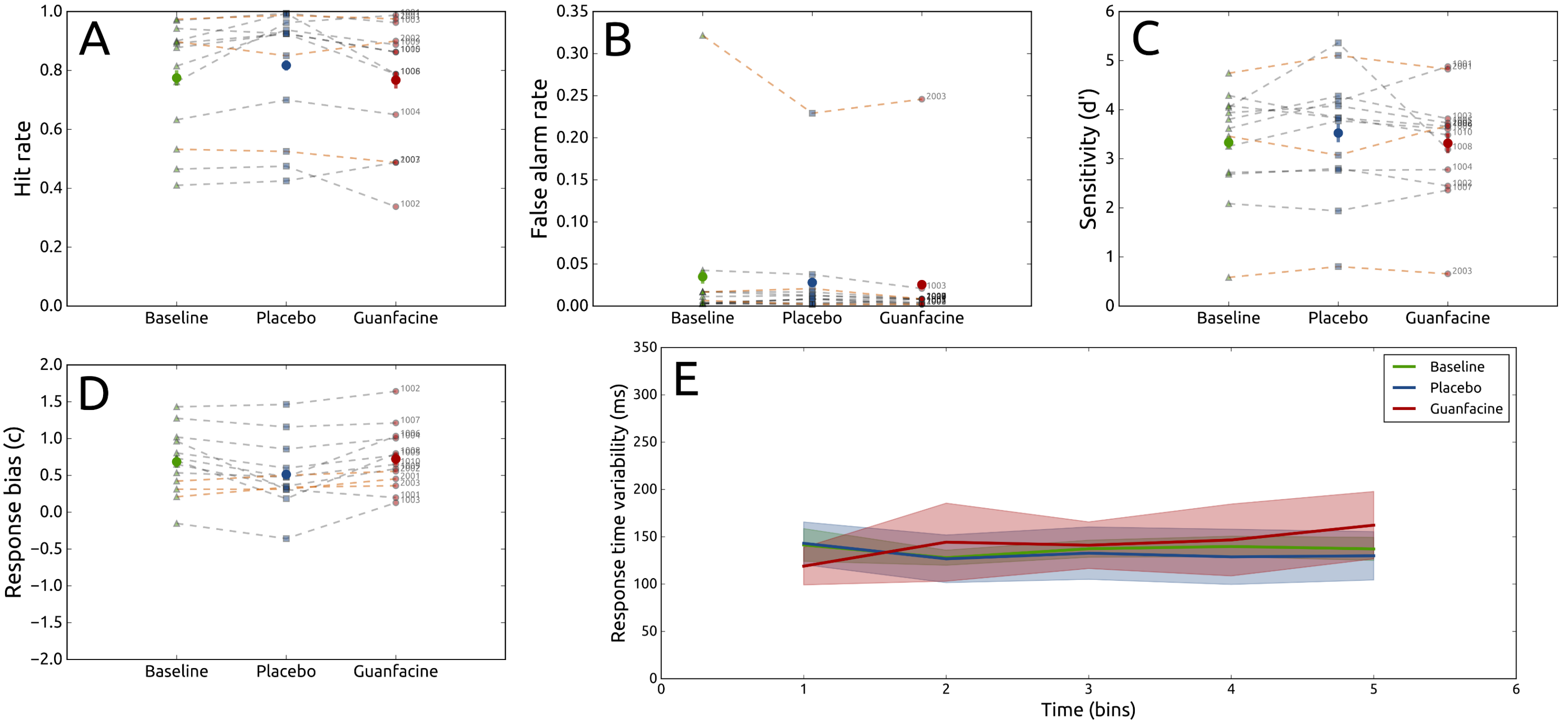

Supplementary Figure 3 – *Signal-detection metrics in a sustained attention task: Hit rate (A), False alarm rate (B), sensitivity d' (C), and response bias c (D), as well as response time variability over time (E) in the baseline (green) condition, and after placebo (blue) or guanfacine (red) administration. Solid horizontal lines indicate the mean, and error bars indicate within-participant 95% confidence intervals. Each dot represents a participant. Each set of connected dots represents a participant (grey for patients with cortical frontal involvement, and orange for without).*
